# Fertility Rate and Assessment of the Cytoprotective Capacity of Various Types of *Holothuroidea* Extracts on Spermatozoa

**DOI:** 10.3390/vetsci9040189

**Published:** 2022-04-15

**Authors:** Alicja Kowalczyk, Elżbieta Gałęska, Anna Szul, Katarzyna Łącka, Anna Bubel, Jose P. Araujo, Riaz Ullah, Marcjanna Wrzecińska

**Affiliations:** 1 Department of Environmental Hygiene and Animal Welfare, Wrocław University of Environmental and Life Sciences, Chełmońskiego 38C, 51-630 Wrocław, Poland; 113226@student.upwr.edu.pl (E.G.); anna.bubel@upwr.edu.pl (A.B.); 2 Malopolska Biotechnic Centre Ltd., 36-007 Krasne, Poland; a.szul@mcb.com.pl (A.S.); k.lacka@mcb.com.pl (K.Ł.); 3 Mountain Research Centre (CIMO), Instituto Politécnico de Viana do Castelo, Rua D. Mendo Afonso, 147, Refóios do Lima, 4990-706 Ponte de Lima, Portugal; pedropi@esa.ipvc.pt; 4 Department of Chemistry, Government College Ara Khel, Kohat 26000, Khyber Pakhtunkhwa, Pakistan; afridiriaz@yahoo.com; 5 Department of Pharmacognosy, College of Pharmacy, King Saud University, Riyadh 12234, Saudi Arabia; 6 Department of Ruminant Science, West Pomeranian University of Technology, Szczecin Klemensa Janickiego 29, 71-270 Szczecin, Poland; marcjanna.wrzecinska@zut.edu.pl

**Keywords:** reproduction, bioextract, quality, sperm, cryopreservation, sea cucumber

## Abstract

For years, compounds of natural origin have been the subject of extensive biomedical research due to very interesting, new ingredients potentially useful for various pharmaceutical, medical and industrial applications. The therapeutic properties and healing benefits of sea cucumbers may result from the presence of numerous, biologically active ingredients. Sperm subjected to processing and subsequent storage at low temperatures experience a number of damage, including the loss of the integrity of the cytoplasmic membrane, DNA and acrosome defragmentation. Therefore, the aim of this experiment was to investigate the cytoprotective potential of sea cucumber extract against cryopreserved sperm and semen fertility rate. Commercially available sea cucumber extract was taken from the cellulose shell, then 790 mg of powder was weighed out and placed in 3 glass tubes containing, respectively: 10 mL of water-glycerin solution (WG), water-ethanol (EC), glycerin-ethanol (GE), glycerin-DMSO (DG). Tubes were mixed with vortex for 3 min, then placed in a water bath and incubated for 16 h at 40 °C. Six simmental bulls, 3 years old, of known health status were used for the experiment. Semen was collected from each male once a week (for 18 weeks) using an artificial vagina. After an initial assessment of semen quality, the ejaculates were pooled to eliminate individual differences between males, then diluted to a final concentration of 80 × 10^6^ sperm/mL with a commercial extender (Optixcell, IMV, L’Aigle, France) and divided into 16 equal samples. Control (C) without additive, the test samples contained 2, 4, 6, 8 and 10 µL WG, 2, 4, 6, 8 and 10 µL WE, 2, 4, 6, 8 and 10 µL GE, 2, 4, 6, 8 and 10 µL DG. Semen was frozen/thawed and assessed for motility, viability, DNA defragmentation, mitochondrial membrane potential and acrosome integrity. It was shown a positive effect of water-glycerin (WG) and glycerine-ethanol (GE) extracts on the efficiency of sperm preservation at low temperatures. Established that, depending on the type of prepared extract, the sea cucumber can have both cytoprotective (WG, GE, WE) and cytotoxic (DG) effects. Moreover, too high concentrations of the extract can adversely affect the sperm in terms of parameters such as viability, motility, mitochondrial potential, and the integrity of the acrosome or DNA of cells. The present study, thanks to the use of model animals to study the cytoprotective potential of the sea cucumber extract, proves that it can be a potential candidate for use in semen cryopreservation technology to improve the efficiency of storage at low temperatures. Further research is needed to optimize the composition of individual types of extracts and their effect on sperm. The highest effectiveness of female fertilization was observed when doses from GE groups (2 and 4) were used for insemination. The results of this analysis prove that the addition of the tested extract may improve the fertilization efficiency.

## 1. Introduction

For years, compounds of natural origin have been the subject of extensive biomedical research due to very interesting, new ingredients potentially useful for various pharmaceutical, medical and industrial applications. The discovery of an impressive range of medicinal functions of sea cucumber, among others, in the field of kidney detoxification, treatment of stomach ulcers, supporting the treatment of respiratory system dysfunctions and accelerating wound healing resulted in a dynamic increase in the interest of researchers in the almost unlimited use of these organisms [1].

The therapeutic properties and healing benefits of *Holothuroidea* (sea cucumbers) may result from the presence of numerous, biologically active ingredients [2]. So far, e.g., peptides, collagen, polysaccharides, fatty acids, nortriterpene and triterpene glycosides, saponins and glycosaminoglycans (chondroitin and fucan sulphates), which are responsible for antioxidant, anticancer and anticoagulant properties of sea cucumber reported in various studies [3,4,5,6]. In addition, sea cucumbers are a great hope in the field of biomedicine due to their demonstrated regenerative capacity (they show the regenerative effect of tissues and organs within a few months) [7].

The aim of the semen cryopreservation is to obtain as many live, morphologically normal sperm as possible, but this process impairs the functions of these gametes [8]. During the process, oxidative and osmotic stress occurs, which results in the production of reactive oxygen species (ROS) [9,10]. Sperm subjected to processing and subsequent storage at low temperatures experience a number of damage, including the loss of the integrity of the cytoplasmic membrane, DNA and acrosome defragmentation [11], which results in a reduction in the viability and motility of sperm cells [11,12]. The reduced quality of semen affects the effectiveness of its use in assisted reproduction (alternative insemination). Bull semen is highly sensitive to cryopreservation, which reduces the viability and motility of sperm after thawing. It is important to evaluate the fertilizing capacity of the sperm in order to eliminate sperm of poor quality. The fertilizing capacity of sperm is determined by key parameters such as concentration, morphology, and sperm motility. Of these parameters, sperm motility and morphology are the most correlated with fertility [13]. The progressive movement of bull’s sperm is crucial to the movement of male gametes in the female reproductive system and to the ability to fertilize. Sperm with high progressive motility are characterized by a greater ability to fertilize the oocyte than sperm with low progressive movement, which may be due to the fact of successfully penetrate the cumulus cells around the oocyte [13,14]. Bull sperm are susceptible to oxidative damage due to the structure of the membrane, which is rich in easily peroxidized polyunsaturated fatty acids, which negatively affects the integrity of the membrane [10,15]. The intact structures of the sperm membrane and acrosomes are necessary for the process of sperm capacitation, the acrosomal reaction and for the fertilization of the oocyte [16]. During semen cryopreservation, changes in the acrosome membrane are observed, including membrane loss compared to non-frozen semen [15,17]. Cryoprotection of sperm acrosomes consists mainly in disintegration of acrosomes through damage to the acrosomal membrane. These changes are mainly due to the formation of ice crystals during the freezing process. Also, osmotic stress can cause changes in the lipid membrane and sperm membrane proteins, which can lead to morphological changes, damage to the membrane continuity and reduced sperm viability [18]. Damaged sperm after cryopreservation, compared to intact sperm, is characterized by destabilization of the membrane due to low temperature and high salt concentration, while the destabilization of the acrosome membrane leads to membrane rupture, folding, or the formation of bubbles in the acrosome membrane. Such acrosome defects are common in bull sperm. maintaining the correct structure of the acrosome, i.e., uninterrupted, unbroken membrane, is important for fertility. Also in semen after cryopreservation, a higher concentration of calcium and the release of acrosomal enzymes, hyaluronidases, and acrosin, which participate in the acrosome reaction, were noticed [18,19,20]. Bull semen during the cryopreservation process shows changes similar to capacitation (cryocapacitation), which consist of phospholipid redistribution, removal of cholesterol, membrane destabilization and calcium influx. This process is considered unfavorable because it results in a reduced quality of cryopreserved semen [10]. In order to increase protection against cryopreservation pf sperm cells, new techniques and methods pf cryopreservation are sought, consisting in adding antioxidants, proteins or extracts to the medium [15]. It has been shown that *Holothuroidea* extract has antioxidant properties, and the addition of these extract to semen before cryopreservation has a protective effect on sperm, preserving their viability and improving the fertilization capacity of cryopreserved semen [9,21].

Therefore, the aim of this experiment was to investigate the cytoprotective potential of sea cucumber extract against cryopreserved sperm and semen fertility rate.

## 2. Material and Methods

Six Simmental bulls, 3 years old, of known health status were used for the experiment. The animals were kept and fed equally. The bulls were kept at the insemination station (Malopolska Biotechnic Centre Ltd., Kraków, Poland) throughout the experiment. The experiment was repeated 18 times.

### 2.1. Preparation of Macerate from Dried Sea Cucumber Extract

Commercially available sea cucumber extract (chondroitin sulfate) *A. japonicus* (Swanson, Fargo, ND, USA) was taken from the cellulose shell, then 790 mg of powder was weighed out and placed in 3 glass tubes containing, respectively: 10 mL of water-glycerin solution (50%/50% *v*/*v*) (WG), water-ethanol (50%/50% *v*/*v*) (EC), glycerin-ethanol (50%/50% *v*/*v*) (GE). Tubes were mixed with vortex for 3 min, then placed in a water bath and incubated for 16 h at 40 °C. pending further analysis.

### 2.2. Preparation and Initial Analysis of Sperm

Semen was collected from each male once a week (for 18 weeks) using an artificial vagina and a hydraulic phantom. The semen (in a graduated tube) was placed immediately after collection in a water bath (temp. 36 °C), where it awaited the preliminary evaluation.

Initial assessments included mass motility, total motility, and sperm viability. Samples showing >50% viable and motile cells were qualified for further procedures.

### 2.3. Supplementation with an Extract of Sea Cucumber

After an initial assessment of semen quality, the ejaculates were pooled to eliminate individual differences between males, then diluted to a final concentration of 80 × 10^6^ sperm/mL with a commercial extender (Optixcell, IMV, L’Aigle, France) and divided into 16 equal samples. Control (C) without additive, the test samples contained 2, 4, 6, 8 and 10 µL WG, 2, 4, 6, 8 and 10 µL WE, 2, 4, 6, 8 and 10 µL GE, 2, 4, 6, 8 and 10 µL DG respectively. After combining with the extract, the samples were gently mixed and subjected to detailed analysis.

Semen was automatically packed (Bloc Machine FIN, IS 4, IMV Technologies, L’Aigle France) into polyvinyl chloride (PVC) straws (0.25 mL) (Biovet, France) which were filled and equilibrated for 3.5 h at 4 °C. After equilibration, the straws were frozen in liquid nitrogen vapor using a computer controlled automatic freezer from 4 °C to −15 °C at the rate of −3 °C/min and from −15 °C to −80 °C at the rate of −10 °C/min (IMV Technologies, L’Aigle, France).

After reaching −80 °C, semen straws were plunged into liquid nitrogen and packaged in plastic goblets for 24 h of storage in the liquid nitrogen container. The straws were thawed in a water bath at 38 °C for 20 s and then were examined to evaluate the quality after thawing.

### 2.4. Assessment of Sperm Viability

The double stain SYBR-14 with propidium iodide (L-7011 LIVE/DEAD Sperm Viability Kit; Invitrogen, Molecular Probes, Barcelona, Spain) using flow cytometer was applied (CytoFlex Beckman Coulter, B3-R1-V0, California, CA, USA). For this purpose, 50 µL of thawed semen was measured (37 °C for 20 s) and 940 µL NaCl (0.9%) and 5 µL SYBR14 were added. The whole was thoroughly mixed and then incubated (36 °C for 10 min) without light access. Subsequently, 5 µL of PI was remixed and incubated 3 min without light, followed by a test [22].

### 2.5. Assessment of Sperm Motility

Mass motility was examined in 20 µL of semen which was placed on a prewarmed slide without any cover slip and analyzed under microscope (Nikon E200, China) equipped with phase-contrast optics (100×) [10]. The mass motility was scored into 4 scales: + no motion, ++ free spermatozoa moving without forming any waves, +++ vigorous movement with moderately rapid waves, ++++ very rapidly moving waves.

Total sperm motility and progressive movement were examined using a Sperm Class Analyzer (SCA, version 5.1, Microptic, Barcelona, Spain), a light microscope (Nikon Eclipse E200). Just prior to analysis, semen was diluted 1:10 in a warm (25 °C) physiological solution (sodium chlorate 0.9%). Then 2 μL of the prepared sample was placed in a Leja 4 analysis chamber (Leja Products B.V., Nieuw-Vennep, Holland) of a thickness of 20.0 μm. The slide was placed on a stage warmer (38 °C). Minimum 500 cells were evaluated, and depending on sperm concentration, five analyses were performed per sample [23].

### 2.6. Assessment of Acrosome Integrity

Acrosome integrity was evaluated by propidium iodide (PI) and fluorescein isothiocyanate-conjugated Pisum sativum agglutinin (FITC-PSA) respectively. This association divides sperm populations into two groups: intact acrosome (IA), damaged acrosome (DA). The procedure was performed with 200,000 cells diluted in SP-Talp, stained with PI (0.5 mg/mL NaCl 0.9%) and FITC-PSA (FITC-PSA L-0770, Sigma, St. Louis, MI, USA, 100 μg/mL in sodium azide NaN 3 solution at 10% in DPBS). Samples were analyzed by flow cytometry after 10 min. All other procedures were performed as previously described [22].

### 2.7. Assessment of the Mitochondrial Membrane Potential

Mitochondrial membrane potential was evaluated by JC-1 probe (5,5′,6,6′-tetrachloro-1,1′,3,3′-tetraethyl-benzimidazolylcarbocyanine chloride, (Invitrogen, Eugene, OR, USA). This probe emits green fluorescent at low (LMP) and medium (MMP) mitochondrial potential or red-orange fluorescent at high potential (HMP). The procedure was performed with 200,000 cells diluted in SP-Talp and stained with JC-1 (76.5 μmol/L in DMSO). Samples were analyzed by flow cytometry after 10 min. All other procedures were performed as previously described [22].

### 2.8. Evaluation of Sperm DNA Fragmentation

To further analyse the sperm DNA integrity, chromatin susceptibility to acid-induced denaturation in situ was assessed. The chromatin instability was then quantified by flow cytometric method (CytoFlex Beckman Coulter, B3-R1-V0, China) using Sperm Chromatin Structure Assay (SCSA) test. The samples were thawed in a water bath (26 °C for 30 s). Thirteen microliters of semen and 487 mL of NaCl (0.9%) were placed in a glass tube on ice. Fifty microliters of thus prepared mixture was moved to the second tube on ice and 100 mL of acid detergent solution (0.08 M HCl, 0.15 M NaCl, 0.1% *v*/*v* Triton X-100, pH 1.2) was added. After exactly 30 s (without a light), 300 mL of acridine orange (AO)-staining solution [6 lg AO (chromatographically purified) (Polysciences, Inc., Warrington, PA, USA) per mL citrate buffer (0.037 M citric acid, 0.126 M Na_2_HPO_4_, 1.1 mM EDTA disodium, 0.15 M NaCl, pH 6.0] was added. The prepared sample was incubated 3 min on ice (without a light) then examined using flow cytometry method; 5000 spermatozoa were evaluated in each sample [23].

### 2.9. Assessment of Sperm Fertility (In Vivo)

Based on in vitro analysis, for insemination, frozen/thawed semen from Control, WG (2 and 4) and GE (2, 4 and 6) groups was used. A total of 60 inseminations (10 repetitions per group) were performed 24 h after the onset of behavioral symptoms of heat. The insemination was carried out by the same trained veterinarian. Only cows of known health status, with no reproductive burden, with a regular oestrus cycle and characteristic oestrus symptoms, were intended for artificial insemination. The animals were kept in the same conditions and fed the same. Pregnancy was diagnosed by rectal examination and digital transrectal ultrasound (DUS 60, EDAN, Shenzhen, China) at least 40 days after insemination.

### 2.10. Statistical Analysis

Data are presented as mean standard error of the mean (SEM). Analysis of variance (ANOVA) was used to assess differences among stages of sea cucmber extract supplementation on all the semen characteristics. When the F ratio was significant (*p* < 0.05), Duncan’s multiple range test was used to compare treatment means. Statistical analysis of the results was performed using Statistica 12.0 (StatSoft, Kraków, Poland). The in vitro experiment was repeated 18 times. The data on in vivo fertility rates were analyzed using the chi-square test.

## 3. Results

Table 1 shows the results of sperm viability analysis before and after the freezing/thawing process.

In the GE (2, 4, 6 8, and 10) and WE (2, 4, 6, 8 and 10) groups, significantly higher sperm viability (*p* < 0.05) was observed compared to the control group. The highest percentage of live sperm was observed in the GE6 group (78%). In turn, in groups DG2, DG4, DG6, DG8 and DG10, a significant decrease in live sperm was observed (*p* < 0.05) in the samples analyzed on average of approximately 6%. A similar trend was observed in cryopreserved ejaculates, the DG2, DG6, DG8 and DG10 groups had the lowest level of sperm survival after thawing, and the results were statistically significantly lower (*p* < 0.05) compared to the other groups studied. The samples marked as groups WG (2, 4, 6, 8, 10), GE2 and WE4 presented the percentage of viable sperm at a significantly higher level than the control (*p* < 0.05).

Table 2 shows the results of the analysis of progressive sperm motility before freezing and after thawing. The results of progressive movement (in fresh semen) in groups WE4, WE10, DG2, DG4, DG6, DG8 and DG10 show a significant downward trend (*p* < 0.05) downward trend. In the DG10 group, the mean value of this parameter was 46.5% and was lower by 7.33% compared to the control group (53.83%). On the other hand, in the GE2 and WG2 groups, the value of this parameter was the highest and amounted to an average of 55.22% and 54.69% (without statistically significant differences in relation to the control (*p* < 0.05)). After analyzing the progressive movement of cryopreserved sperm, a drastic decrease in the mean value of this parameter was observed in the DG4, DG6, DG8 and DG10 groups by 6.36%, 8.61%, 16.64% and 17.93%, respectively. In the GE10 group, a low percentage of progressive movement sperm was also observed (33.19%). Significant differences (*p* < 0.05) in terms of the parameter analyzed were observed in the GE2 group, where the percentage of progressive sperm after thawing was significantly higher and was 43.92%.

Table 3 shows the results of the sperm acrosome integrity analysis after thawing. When analyzing the IA sperm population, it was observed that the best results in terms of this index were obtained in the groups WG2 (47.03%), WG4 (46.90%), GE2 (46.19%) and GE4 (46.71%), these results differed significantly (*p* < 0.05) in relation to the control group. In the DG4, DG6, DG8 and DG10 groups, a significant (*p* < 0.05) decrease (*p* < 0.05) in sperm with intact acrosome was observed on average by 7.6%, 6.75%, 8.18 and 9.82% compared to the control group. The higher concentration of sea cucumber extract in the WG (8 and 10) and GE (8) groups did not improve the IA parameter or significantly worsen it. The highest percentage of the DA sperm population was observed in the DG2, DG4, DG6, DG8 and DG10 groups; it was on average more than 60% of sperm cells.

The results for low, medium and high potential of frozen/thawed sperm are presented in Table 4. In terms of the HMP parameter analyzed in groups WG2, GE2, GE8, WE2, WE4 and DG4, a significant (*p* < 0.05) increase in potential was observed compared to the control group by 2.36%, 3.38%, 3.32%, 3.44% and 3.2%, respectively. The percentage of sperm with low mitochondrial potential was the highest in the DG8 and DG10 groups, this parameter increased significantly by 5.51% and 9.64%, respectively, compared to the control (83.34%). The lowest percentage of sperm from MMP was obtained in the groups WE10 (30.21%), DG2 (29.99%), DG4 (33.22%), DG6 (19.89%), DG8 (18.46%) and DG10 (14.08%).

In this study, it was observed (Table 4) that in the WG2, WG4, WG6, GE2 and GE4 groups, the percentage of spermatozoa with damaged DNA decreased significantly (*p* < 0.05) by 2.79%, 2.74%, 2.65%, 3.19% and 2.98%, respectively, compared to the group. in the control (10.2%). The opposite trend was observed in groups DG2, DG4, DG6, DG8, and DG10, where an increase in the percentage of sperm with damaged DNA was observed. In the DG10 group, a significant (*p* < 0.05), approximately 3% increase in DNA damage was observed in the sperm population analyzed compared to the control (10.2%). The best results in terms of DNA protection against damage were obtained in the GE2 group (7.01%).

Table 5 shows the results of the effectiveness of fertilization of females with frozen/thawed semen. Based on the previously obtained results of the in vitro semen quality analysis, doses from the Control, WG (2 and 4) and GE (2, 4 and 6) groups were assigned for insemination.

The Control, WG2 and GE6 groups did not differ in terms of the analyzed trait, as the fertility level of the animals in these groups was identical and amounted to 70%.

The lowest fertility index was observed for females inseminated with semen from the WG4 group (60%), it was statistically significantly lower (*p* < 0.05) than the level obtained in the control, WG2, GE6 groups and highly significantly lower (*p* < 0.01) than the index for animals from the groups GE2 and GE4. The animals inseminated with sperm from the GE4 group (90%) showed the highest level of fertility. The level of the analyzed trait turned out to be statistically significantly significantly higher (*p* < 0.01) compared to the other groups.

## 4. Discussion

The purpose of this study was to evaluate the cytoprotective capacity of various types of sea cucumber extracts on sperm. In this study, for the first time the parameters of cryopreserved sperm were assessed in the presence of various types of sea cucumber extracts.

Due to the preservation fact that sperm in the process of preservation at low temperatures experience many negative effects, including damage to the cytoplasmic membrane, lower overall motility and motility parameters, acrosome damage, and a decrease in mitochondrial potential [24], it is necessary to optimize the environment in which cells are stored.

The multifaceted potential of the sea cucumber as a product that can be used in animal and human biomedicine prompted us to try to use it as a cytoprotector. Sea cucumber extracts have been shown to contain bioactive compounds as antioxidants such as triterpene glycosides as inhibitors of degenerative diseases due to free radicals such as heart disease and cancer [25,26]. In addition, its physiological and nutraceutical benefits have been proven, especially to improve sexual performance [2].

In our study, we showed a positive effect of water-glycerin (WG) and glycerine-ethanol (GE) extracts on the efficiency of sperm preservation at low temperatures. Moreover, we were able to establish that, depending on the type of prepared extract, the sea cucumber can have both cytoprotective (WG, GE, WE) and cytotoxic (DG) effects. We also came to the conclusion that too high concentrations of the extract may adversely affect the sperm in terms of parameters such as viability, motility, mitochondrial potential, and the integrity of the acrosome or DNA of cells. It seems that the dynamic change in the mode of action of extracts is due to the presence of various types of bioactive ingredients in the sea cucumber, including toxins [27].

The positive effect of the sea cucumber extract reported in this study is probably due to the specific composition of the sea cucumber itself. Both polysaccharides [28], saponins [29], polyphenols, and cholesterol [30] have been shown to have a beneficial effect on sperm. The health-promoting properties of sea cucumber result from the content of many biologically active substances [30,31]. It is known that sea cucumbers are rich in polysaccharides, such as glucosamines, galactose, or glucuronic acid, which have antioxidant properties. Moreover, flucoidate and fucosylated chondroitin sulfate, as well as polyphenols, phospholipids, cerebrosides, and flavonoids also exhibit such activity [9]. Sea cucumber proteins are mainly obtained from the body walls, which are rich in glycine, glutamic acid and arginine, which stimulate the activation and proliferation of natural killer cells, as well as the release of interleukon-6 (IL-6) and B cells necessary for the intensification of phagocytosis. Due to the content of these amino acids, sea cucumbers contribute to the modulation of immunity. In addition to the therapeutic use of sea cucumbers, they are also used as an aphrodisiac, improving sexual performance [2]. Saponins have antimicrobial, anti-inflammatory, anti-cancer and antioxidant properties. The antioxidant activity of these compounds contained in sea cucumbers is based on the improvement of superoxide dismutase (SOD) activity, removal of reactive oxygen species, inhibition of lipid peroxidation. According to Timar et al. (2022) [32], saponins can increase sperm count, reduce the percentage of immature and DNA-damaged sperm due to oxidative stress [32]. Moreover, sea cucumbers are rich in various minerals and vitamins, including zinc, which is important in DNA organization, stabilization of cell membranes, the process of spermatogenesis, and its level is correlated with sperm motility [14,33]. Low zinc levels result in testosterone production. Zinc deficiency in the male reproductive system may be associated with insufficient spermatogenesis, infertility, or negatively affect sperm. Its concentrations is related to the concentrations of sperm in the semen [33].

Poor sperm motility is one of the leading causes of male infertility and assisted reproductive technology (ART) fertilization failure. The results of our research indicate that some types of extracts of WG (2, 4) and GE (2, 4, 6) not only maintain sperm motility at the correct level before freezing, but also have a positive effect on the percentage of progressive motility of sperm cells after cryopreservation.

Moreover, we believe that it is related to the positive influence of the types of extracts studied on mitochondria, which we demonstrated. Mitochondria, whose sperm counts are limited in the midpiece, play a vital role in sperm function by providing energy. This energy is necessary for sperm cells to carry out the cellular processes involved in fertilization success, such as motility, hyperactivation, capacitation, and acrosome response. Therefore, an integral aspect of maintaining normal sperm cell function in terms of quality and fertilization capacity is mitochondrial potential (MMP) [34,35]. Mitochondrial functions are crucial to the energy status and motility of sperm through the production of energy (ATP) that drives flagella and causes cell movement [17] The process of cryopreservation of semen influences the formation of cryoprotection of sperm mitochondria in the form of distortions of these organelles or by changing the potential of the mitochondrial membrane of bulls [18]. There is a close relationship between sperm motility and the functional state of the gamete mitochondria. It has been shown that sperm with a lower mitochondrial membrane potential are associated with male infertility. Oxidative stress also adversely affects mitochondrial activity and impaired sperm motility [16]. In the present experiment, it was shown that WG2 and GE2 extracts help sperm maintain a high mitochondrial potential after thawing.

Regardless of semen quality, concurrent testing of both MMP and sperm DNA fragmentation was found to be better predictors of natural conception in infertile men [36]. For this reason, the present study evaluated the effect of individual extract types on DNA integrity and demonstrated that both glycerol, glycerol-ethanol, and water-ethanol extracts of sea cucumbers effectively protect sperm DNA from damage during freezing/thawing.

The highest effectiveness of female fertilization was observed when doses from GE groups (2 and 4) were used for insemination. The results of this analysis prove that the addition of the tested extract may improve the fertilization efficiency. The observed higher dose efficacy in these groups compared to the control may be related to the previously proven better sperm cryostability during the freezing process. Better sperm viability and motility (after thawing compared to control) combined with reduced cell DNA damage could contribute to better effects of female insemination.

Obtaining biologically active compounds from squirrels takes place in several stages, which are pre-treatment, extraction, and purification. The most common pre-treatment methods are freeze drying, drying, ultrasonic drying, and red radiation, which reduce the loss of nutritional value. Then, extraction is carried out, most often with organic solvents, and purification is mainly carried out by means of chromatography or ultrafiltration in order to remove specific protein or polysaccharide impurities [37]. Fresh, air-dried and freeze-dried guts of sea cucumbers were analyzed for biological properties and nutrient content. It was shown that the guts of sea cucumbers subjected to the drying and freeze-drying process were characterized by a similar level of ash, lipids, including fatty acids and polyunsaturated fatty acids, proteins, as well as fresh guts. This indicates the high potential of the drying and freeze-drying process to preserve nutritional and biologically active values [38]. On the other hand, studies on pomegranate fruit skins showed higher concentrations of phenols, tannins and flavonoids after freeze-drying, which translated into antibacterial and antioxidant activity [39]. Many studies have confirmed the antioxidant properties of extracts obtained by freeze-drying [27,38]. In turn, it was shown that sea cucumber extract contains more phenols compared to organic extracts [2]. It was shown that hydrated samples, especially those containing internal organs, showed greater antioxidant activity than those from fresh sea cucumber samples. In addition, the hydrolyzate of the syringe gelatin prevents oxidative damage to the rabbit’s liver and mitochondria, which at the same time has a better effect than vitamin E, which is an antioxidant. On the other hand, among sea cucumber extracts, extracts obtained with ethyl acetate showed the highest antioxidant activity compared to water extracts. It also confirms that sea cucumbers can be an addition to the diet [37,40].

The content of the above ingredients in sea cucumber makes it a promising biomedical agent for use in assisted reproduction in both humans and animals.

## 5. Conclusions

The present study, thanks to the use of model animals to study the cytoprotective potential of the sea cucumber extract, proves that it can be a potential candidate for use in semen cryopreservation technology to improve the efficiency of storage at low temperatures. Further research is needed to optimize the composition of individual types of extracts and their effect on sperm. Moreover, the results of this analysis prove that the addition of the tested extract may improve the fertilization efficiency.

## Figures and Tables

**Table 1:** Effect of various types of sea cucumber extract on viability and total motility of spermatozoa before and after cryopreservation. Explanations: a–c—means with different superscript letters in the same column differ significantly at *p* < 0.05.

Group	Before Freezing		After Freezing	
	Live Cells [%]	Total Motility [%]	Live Cells [%]	Total Motility [%]
Control	72.90a ± 3.01	73.33a ± 4.16	44.93a ± 3.07	55.31a ± 3.16
WG 2	74.96ab ± 2.93	74.07a ± 4.22	52.05b ± 2.17	57.13a ± 3.49
WG 4	75.95ab ± 3.22	73.00a ± 3.88	50.94b ± 2.69	54.17ab ± 3.02
WG 6	75.00ab ± 2.77	71.94a± 2.21	51.99b ± 2.41	49.99ab ± 2.94
WG 8	74.91ab ± 2.64	72.22a ± 3.04	54.02b ± 3.07	49.98ab ± 2.91
WG 10	75.08ab ± 2.00	72.38a ± 3.65	51.15b ± 2.94	51.95ab ± 2.70
GE 2	75.93b ± 1.90	76.76a ± 3.71	50.00b ± 3.00	56.27a ± 3.11
GE 4	76.11b ± 1.92	75.15a ± 2.92	43.37a ± 2.29	52.04ab ± 3.90
GE 6	78.28b ± 1.95	74.91a ± 2.71	43.08a ± 1.91	52.91a ± 3.44
GE 8	76.44b ± 2.00	74.43a ± 3.19	45.39ab ± 2.14	49.90ab ± 3.56
GE 10	77.01b ± 2.20	74.41a ± 4.04	41.93c ± 2.91	48.37b ± 2.95
WE 2	77.66b ± 1.98	73.00a ± 4.27	43.96ab ± 2.88	52.19ab ± 2.71
WE 4	76.93b ± 2.33	72.81a ± 3.80	49.02b ± 2.91	53.36ab ± 3.09
WE 6	78.71b ± 1.66	72.90a ± 3.04	43.44a ± 2.68	48.61b ± 3.12
WE 8	78.04b ± 1.72	73.09a ± 2.12	44.81ab ± 2.55	47.47b ± 4.07
WE 10	78.81b ± 2.13	72.99a ± 3.06	43.01a ± 3.03	46.89b ± 3.24
DG 2	71.01c ± 1.24	72.02a ± 3.13	43.24ab ± 2.63	46.78b ± 3.41
DG 4	71.55c ± 1.27	72.16a ± 2.97	52.11b ± 2.22	45.20b ± 3.97
DG 6	71.06c ± 2.10	72.21a ± 3.61	38.88c ± 3.12	11.67b ± 3.10
DG 8	65.94c ± 1.44	71.96a ± 3.54	42.32ac ± 3.10	42.91b ± 3.44
DG 10	61.99c ± 2.42	71.90a ± 3.25	42.70ac ± 2.97	42.01b ± 2.54

**Table 2:** Effect of various types of sea cucumber extract on progressive motility of spermatozoa before and after cryopreservation. Explanations: a–d—means with different superscript letters in the same column differ significantly at *p* < 0.05.

Group	Progressive Motility [%]	
	Before Freezing	After Freezing
Control	53.83a ± 2.22	34.92b ± 3.71
WG 2	54.69a ± 2.61	38.88ab ± 3.66
WG 4	54.00a ± 2.54	39.02ab ± 3.50
WG 6	49.99ab ± 2.77	34.89b ± 4.07
WG 8	50.00ab ± 2.91	34.61b ± 3.92
WG 10	50.04ab ± 2.00	35.06ab ± 2.91
GE 2	53.00a ± 1.61	43.92a ± 3.10
GE 4	53.06a ± 2.04	35.91ab ± 3.27
GE 6	52.39a ± 2.31	35.02ab ± 3.16
GE 8	50.52a ± 2.66	34.94b ± 3.54
GE 10	49.04ab ± 2.80	32.00bc ± 4.04
WE 2	49.11ab ± 1.95	35.15ab ± 4.11
WE 4	46.10b ± 2.77	37.67ab ± 3.88
WE 6	53.31a ± 3.09	32.24bc ± 3.67
WE 8	49.96ab ± 2.10	30.30bc ± 3.51
WE 10	47.71b ± 2.96	29.97c ± 4.12
DG 2	47.12b ± 3.12	30.01c ± 4.21
DG 4	45.04b ± 3.24	28.56c ± 3.74
DG 6	46.22b ± 3.65	26.31c ± 3.51
DG 8	46.00b ± 4.01	18.28cd ± 5.06
DG 10	46.50b ± 2.67	16.99d ± 5.19

**Table 3:** Effect of various types of sea cucumber extract on the integrity of the sperm acrosome after cryopreservation. Explanations: a–d—means with different superscript letters in the same column differ significantly at *p* < 0.05.

Group.	Acrosome Integrity [%]	
	Live Intact Acrosome	Dead Intact Acrosome
Control	42.93b ± 1.90	54.47b ± 2.16
WG 2	47.03a ± 1.81	49.96c ± 2.53
WG 4	46.90a ± 1.77	49.91c ± 2.76
WG 6	42.27b ± 1.51	54.37bc ± 2.19
WG 8	42.21b ± 1.94	53.91b ± 2.07
WG 10	41.90b ± 2.02	55.15b ± 2.54
GE 2	46.19a ± 1.66	50.60c ± 1.99
GE 4	46.71a ± 1.61	50.00c ± 2.70
GE 6	46.03a ± 1.14	51.27c ± 2.44
GE 8	41.91bc ± 2.21	55.61b ± 2.74
GE 10	39.87bc ± 2.33	57.09b ±2.81
WE 2	39.00b ± 1.92	55.55b ± 2.82
WE 4	40.05ab ± 1.74	55.26bc ± 1.92
WE 6	38.21bc ± 2.50	57.71b ± 2.17
WE 8	38.07bc ± 2.32	57.00b ± 2.36
WE 10	37.99bc ± 2.39	57.03b ± 2.51
DG 2	36.31c ± 2.89	58.81b ± 1.55
DG 4	35.33d ± 2.68	60.09ab ± 2.00
DG 6	36.18cd ± 2.51	60.00ab ± 1.96
DG 8	34.75d ± 2.80	61.72ab ± 2.04
DG 10	33.65d ± 2.79	62.96a ± 1.90

**Table 4:** Effect of various types of sea cucumber extract on the mitochondrial membrane potential and DNA integrity of sperm after cryopreservation. Explanations: a–e—means with different superscript letters in the same column differ significantly at *p* < 0.05,.

Group	Mitochondrial Membrane Potential			DNA Integrity [%]
	High [%]	Medium [%]	Low [%]	
Control	12.00b ± 0.66	36.96b ± 3.16	77.77b ± 3.55	10.20bc ± 0.42
WG 2	14.36a ± 0.81	40.05ab ± 3.00	76.99bc ± 4.14	7.41d ± 0.31
WG 4	14.41ab ± 0.74	48.80b ± 3.47	77.41bc ± 3.91	7.46d ± 0.29
WG 6	14.20ab ± 1.10	48.51b ± 3.50	74.49c ± 3.62	7.55d ± 0.44
WG 8	14.21ab ± 0.94	47.79b ± 3.12	77.03bc ± 3.54	7.61cd ± 0.56
WG 10	14.20ab ± 0.77	47.57b ± 3.71	78.00b ± 4.16	7.65cd ± 0.79
GE 2	15.38a ± 0.61	42.06a ± 2.99	73.99c ± 3.90	7.01d ± 0.51
GE 4	14.31ab ± 0.92	39.90ab ± 3.55	74.69bc ± 3.76	7.22d ± 0.64
GE 6	14.45ab ± 0.88	39.99ab ± 3.30	73.97c ± 3.20	7.50c ± 0.70
GE 8	15.32a ± 1.00	39.93ab ± 2.61	72.28c ± 4.19	7.49c ± 0.59
GE 10	13.01b ± 0.71	33.14bc ± 4.05	77.99b ± 3.58	7.57c ± 0.82
WE 2	15.44a ± 0.82	37.00b ± 3.92	74.15c ± 3.91	7.42cd ± 0.71
WE 4	15.20a ± 0.95	42.20a ± 3.51	73.91c ± 3.64	7.91c ± 0.52
WE 6	14.17b ± 0.90	35.51bc ± 2.70	77.08b ± 3.22	8.00c ± 0.56
WE 8	14.10b ± 0.75	35.50bc ± 3.51	77.90b ± 4.18	8.29c ± 0.77
WE 10	14.05b ± 0.91	30.21dc ± 4.10	77.96b ± 2.79	8.33c ± 0.72
DG 2	11.02b ± 1.06	29.99dc ± 3.77	79.92b ± 3.20	9.05bc ± 0.84
DG 4	15.07a ± 0.99	33.22c ± 3.45	73.05c ± 4.10	8.90c ± 0.62
DG 6	10.10bc ± 1.10	19.87e ± 4.22	79.00b ± 3.76	11.40b ± 0.51
DG 8	6.50c ± 0.94	18.46e ± 4.54	80.11ab ± 3.55	11.55ab ± 0.63
DG 10	5.12cd ± 0.90	14.08e ± 3.00	87.06a ± 3.41	12.09a ± 0.88

**Table 5:** Fertility rate in cryopreserved semen with *Holothurioidea* extract addition.

Groups	Fertility Rate (%)	Control	WG 2	WG 4	GE 2	GE 4	GE 6
		70	70	60	80	90	70
Control	70	-	ns	*	*	**	ns
WG 2	70	0.9980	-	*	*	**	ns
WG 4	60	0.0311	0.0232	-	**	**	*
GE 2	80	0.0411	0.0312	0.0001	-	**	*
GE 4	90	0.0001	0.0001	0.0001	0.0001	-	**
GE 6	70	0.9786	0.9998	0.4251	0.0411	0.0001	-
chi-square = 19.3, *p*-value							

* significant (*p* < 0.05); ** highly significant (*p* < 0.01); ns- not significant.

## Data Availability

The reporting in the manuscript follows the recommendations in the ARRIVE guidelines [41]. The datasets used during the current study are available from the corresponding author on reasonable request.
